# Rate-Compatible, Bandwidth-Efficient, Low-Density Parity-Check (LDPC) Codes for Aeronautical Telemetry †

**DOI:** 10.3390/e26121045

**Published:** 2024-11-30

**Authors:** Andrew D. Cummins, David G. M. Mitchell, Erik Perrins

**Affiliations:** 1 Klipsch School of Electrical and Computer Engineering, New Mexico State University, Las Cruces, NM 88003, USA; dgmm@nmsu.edu; 2 Department of Electrical Engineering & Computer Science, University of Kansas, Lawrence, KS 66045, USA; esp@ku.edu

**Keywords:** FEC, LDPC codes, telemetry, avionics, IRIG, spectral efficiency, puncturing, shortening, iterative decoding

## Abstract

Low-density parity-check (LDPC) codes form part of the IRIG-106 standard and have been successfully deployed for the Telemetry Group version of shaped-offset quadrature phase shift keying (SOQPSK-TG) modulation. Recently, LDPC code solutions have been proposed and optimized for continuous phase modulations (CPMs), including pulse code modulation/frequency modulation (PCM/FM) and the multi-h CPM developed by the Advanced-Range TeleMetry program (ARTM CPM), the latter of which was shown to perform around one dB from channel capacity. In this paper, we consider the effect of the random puncturing and shortening of these LDPC codes to further improve spectrum efficiency. We perform asymptotic analyses of the ARTM0 code ensembles and present numerical simulation results that affirm the robust decoding performance promised by LDPC codes designed for ARTM CPM.

## 1. Introduction and Motivation

Low-density parity-check (LDPC) codes [1] are a family of forward error correction (FEC) block codes that achieve capacity-approaching performance under low-complexity iterative decoding schemes [2,3]. Efficient LDPC encoder designs have given rise to implementations [4,5] that meet the stringent requirements of low-power transmitters, and LDPC decoding algorithms are well suited to highly parallelized decoder structures that facilitate high-throughput FEC [6,7]. LDPC codes have been successfully deployed for aeronautical telemetry systems that employ continuous phase modulations (CPM), specifically SOQPSK-TG [8]. Recent work outlining techniques for the construction of LDPC codes for the remaining two CPM modulation schemes, PCM/FM and ARTM CPM, have demonstrated performance within one dB of channel capacity in numerical simulations [9,10]. While keeping encoding complexity low, these codes represent the current best FEC performance published for ARTM CPM, and further optimizing these codes for deployment in aeronautical telemetry systems under bandwidth constraints is an open problem.

One remaining challenge is to develop spectrally efficient LDPC coding schemes for CPM that can adapt to the changing conditions of time-varying channels. Coding schemes that can adaptively respond to changing channel conditions without the need for alterations to either their encoder or decoder hardware configurations are known as *rate-compatible* codes [11]. An efficient means of accomplishing this is to omit sending a portion of the encoded information stream, a process known as *puncturing* [11,12]. This technique relies on the strength of the code and the effect of the puncturing pattern on the decoding algorithm to provide sufficient, though diminished, error correction performance without requiring changes to the decoder [13,14,15,16,17,18]. No *a priori* information on punctured bits is shared with the decoder prior to transmission (other than their location), and thus the code rate is increased at the expense of a handicapped decoder. A complementary method, *shortening* [19], follows much the same logic, but certain code symbols are fixed at the encoder and omitted from the transmission, with the decoder knowing only the location of those shortened bits. This modification can be achieved at the decoder by providing known, perfect *a priori* information on shortened bits, improving the overall iterative decoding performance at the expense of a decrease in both the code rate and the dimension. Shortening has been shown to improve the performance of LDPC codes [17,20,21], and an analysis of random shortening was performed in [22]. We assume that by co-implementation of the transmitter and receiver the fixed *locations* of punctured or shortened bits are known by both the encoder and decoder throughout our designs.

### 1.1. Methods and Contributions

In this paper, we use random puncturing and shortening to explore the trade-off between the coding rate and error control performance for some of the LDPC coding schemes proposed in [9,10], enabling the development of spectrally efficient, rate-compatible coding strategies, which require minimal alterations to proven hardware implementations. We follow the notation established in [9,10] by using ARTM0, ARTM1, and ARTM2 to refer to PCM/FM, SOQPSK-TG, and ARTM CPM, respectively. We begin by performing asymptotic analyses of the ARTM0 code ensembles. First, we determine the iterative belief propagation (BP) decoding thresholds of randomly punctured ARTM0 LDPC code ensembles on the binary erasure channel (BEC). We then perform an asymptotic minimum distance analysis to establish that both the mother code and the punctured code ensembles are asymptotically good. Despite the minimum distance growth rates decreasing with the puncturing fraction, we show that the distance to the Gilbert–Varshamov bound shrinks with puncturing. Taken together, the asymptotic analyses indicate that the ARTM0 LDPC code ensembles are good candidates for random puncturing.

We then present numerical simulation results for various puncturing ratios to create effective coding rates in between those previously demonstrated. To isolate the effect of modulation on the LDPC encoder–decoder pair, we present results with both binary phase-shift keying (BPSK) and CPM modulation, highlighting the significant coding gain achieved through the optimized design of the LDPC codes with respect to specific modulation schemes. We observe that CPM decoding feedback is responsible for more than 2 dB of coding gain, illustrating the impact of optimization for that regime. Our results demonstrate that significant gains in spectral efficiency can be obtained for a reasonable degradation in FEC performance. For example, with 5% of the symbols omitted from transmission by puncturing, we are able to increase a code with a rate of R=2/3=0.667 to R=0.701 with less than a 0.2 dB loss at a bit error rate of $10^{-6}$ and with no alterations to the component code or decoder required.

We also provide numerical simulation results for shortened codes showing marked improvements in waterfall performance for codes of sufficient rate and block length. For example, for the ARTM0 mother code with R=4/5 and information block length k=4096, a coding gain of more than 0.2 dB can be obtained with random shortening under the CPM-modulated scheme. Furthermore, interchanging hardware to allow for shortening and puncturing is not necessary, as both techniques utilize overlapping function blocks with few alterations and may be combined for further flexibility when designing rate-comp- atible codes.

### 1.2. Paper Structure

The paper is structured as follows. Section 2 provides the necessary background material, including protograph-based LDPC code construction and the code-modification techniques we will use, namely puncturing and shortening. In Section 3, we perform asymptotic analyses of the randomly punctured protograph-based ARTM0 ensembles to determine their suitability for puncturing from the perspective of BP and minimum distance. Section 4 and Section 5 provide the system models and numerical simulation results obtained for random puncturing and shortening, respectively. Section 6 then provides a brief description of the hardware considerations before some concluding remarks are provided in Section 7.

## 2. Background

### 2.1. Protograph-Based LDPC Codes

An (n,k) LDPC code, with length *n* and dimension *k*, is described as the null space of a parity-check matrix H that has a rank of n−k. We say that an LDPC code is (J,K)-*regular* if it has precisely *J* ones in every column and *K* ones in every row; otherwise, it is called *irregular*. A *protograph* [23], with design rate $R=1-n_{c}/n_{v}$, is a small bipartite graph whose edges connect a set of $n_{v}$ variable nodes to a set of $n_{c}$ check nodes. This structure can be characterized by a *base* biadjacency matrix B, where $B_{x,y}$ is taken to be the number of edges connecting variable node $v_{y}$ to check node $c_{x}$. To form the parity-check matrix H of a protograph-based LDPC code, each non-zero entry in B is replaced by a sum of $B_{x,y}$ non-overlapping M×M permutation matrices, and zero entries are replaced by the M×M all-zero matrix.

**Example** **1.** *The (irregular) ARTM0 protographs [9] are given as*

(1) $$\begin{array}{c} B_{0,1/2}=\left[\begin{array}{cccccccc} 0 & 0 & 0 & 0 & 0 & 0 & 1 & 2\\ 0 & 0 & 0 & 0 & 1 & 1 & 0 & 2\\ 0 & 0 & 1 & 1 & 0 & 0 & 1 & 2\\ 3 & 3 & 2 & 2 & 2 & 2 & 1 & 1 \end{array}\right], \end{array}$$

(2) $$\begin{array}{c} B_{0a,2/3}=\left[\begin{array}{cccccccccccc} 0 & 0 & 0 & 0 & 0 & 0 & 1 & 0 & 0 & 1 & 2 & 1\\ 0 & 0 & 0 & 0 & 0 & 1 & 0 & 0 & 1 & 1 & 1 & 2\\ 0 & 0 & 0 & 0 & 1 & 0 & 0 & 1 & 0 & 0 & 2 & 2\\ 3 & 3 & 3 & 3 & 2 & 2 & 2 & 3 & 3 & 2 & 0 & 1 \end{array}\right], \end{array}$$

(3) $$\begin{array}{c} B_{0b,2/3}=\left[\begin{array}{cccccccccccc} 0 & 0 & 0 & 0 & 0 & 0 & 0 & 0 & 0 & 2 & 1 & 2\\ 0 & 0 & 0 & 0 & 0 & 0 & 0 & 1 & 1 & 1 & 1 & 2\\ 1 & 1 & 1 & 1 & 1 & 1 & 2 & 0 & 0 & 0 & 0 & 0\\ 2 & 2 & 2 & 2 & 2 & 2 & 1 & 2 & 2 & 0 & 1 & 0 \end{array}\right],and \end{array}$$

(4) $$\begin{array}{c} B_{0,4/5}=\left[\begin{array}{cccccccccccccccccccc} 0 & 0 & 0 & 0 & 0 & 0 & 0 & 0 & 0 & 0 & 0 & 0 & 0 & 0 & 2 & 0 & 1 & 2 & 2 & 2\\ 0 & 0 & 0 & 0 & 0 & 0 & 0 & 0 & 0 & 0 & 0 & 0 & 1 & 1 & 1 & 1 & 0 & 2 & 2 & 2\\ 1 & 1 & 1 & 1 & 1 & 1 & 2 & 2 & 2 & 2 & 2 & 2 & 0 & 2 & 0 & 1 & 2 & 1 & 1 & 0\\ 2 & 2 & 2 & 2 & 2 & 2 & 1 & 1 & 1 & 1 & 1 & 1 & 2 & 0 & 0 & 1 & 1 & 0 & 0 & 1 \end{array}\right], \end{array}$$

*following the naming convention of [9] that $B_{0,1/2}$ refers to the ARTM0 LDPC code ensemble of design rate R=1/2 and so on.*

### 2.2. Puncturing Linear Codes

An (n,k) linear code is *punctured* by removing a set of *p* columns from its generator matrix. This procedure effectively reduces the codeword length from *n* to n−p and the *puncturing fraction* $\alpha_{p}=p/n$ can be adjusted to alter the transmission rate to

(5) $$R\left(\alpha_{p}\right)=\frac{R}{1-\alpha_{p}}\;,\;\alpha_{p}\in \left[0,1\right),$$

where R(0)=R is the rate of the mother (unpunctured) code. Puncturing preserves the dimension of the code, achieving the target rate $R(\alpha_{p})$, provided that no two distinct codewords only differ within the *p* punctured symbols. This can be achieved, for example, by restricting punctured symbols to the n−k parity-check symbols of a code. The decoder will estimate both the punctured and transmitted symbols during decoding, and thus the receiver must know the puncturing pattern utilized by the transmitter prior to establishing communication.

### 2.3. Shortening Linear Codes

An (n,k) linear code is *shortened* by keeping only the codewords from the mother code that have zeros at the positions of the *s* shortened bits. This is equivalent to removing the corresponding set of *s* columns from its parity-check matrix. In this paper, we restrict shortening to the *k* information symbols. Shortening will reduce both the dimension and the length of the code. After shortening a linear code with *shortening fraction* $\alpha_{s}=s/n$, the resulting transmission rate is

(6) $$R^{s}\left(\alpha_{s}\right)=\frac{R-\alpha_{s}}{1-\alpha_{s}}=\frac{k-s}{n-s}\;,\;\alpha_{s}\in \left[0,1\right),$$

where $R^{s}\left(0\right)=R$ is the rate of the mother (unshortened) code.

As with puncturing, a code can be shortened randomly or according to a particular pattern. In order to be rate-compatible, we assume that the receiver knows the positions of the shortened symbols and maintains perfect information about those bits in order to estimate the transmitted symbols during decoding.

## 3. Asymptotic Random Puncturing Analyses of ARTM0 Protographs

In this section, we examine the suitability of the ARTM0 protographs for random puncturing. We remark that specific, finite-length graph liftings were optimized in [9] in order to achieve excellent performance with CPM and a two-stage iterative decoder; however, in this section, we assume asymptotic random liftings for the purpose of the analysis of the code ensembles.

### 3.1. BEC Decoding Thresholds of Randomly Punctured ARTM0 Code Ensembles

Consider puncturing a length *n* codeword v for transmission over a BEC with erasure probability ϵ. We assume that a fixed fraction α=p/n of the code symbols are punctured, such that the transmitted codeword $v^{\mathrm{punc}}$ has length $n^{\mathrm{punc}}=\left(1-\alpha \right)\cdot n$. After transmission, the received vector r will contain, on average, $\epsilon \cdot n^{\mathrm{punc}}$ erased symbols and $\left(1-\epsilon \right)\cdot n^{\mathrm{punc}}$ correct symbols. The receiver knows the positions of the punctured and erased symbols and proceeds to decode the overall code of length *n*. From [13], we recall that the BP threshold $\epsilon_{\mathrm{BP}}\left(\alpha_{p}\right)$ of a randomly punctured LDPC code ensemble on the BEC with puncturing fraction $\alpha_{p}$ is given by

(7) $$\epsilon_{\mathrm{BP}}\left(\alpha_{p}\right)=1-\frac{1-\epsilon_{\mathrm{BP}}\left(0\right)}{R}\cdot R\left(\alpha_{p}\right),$$

where $\epsilon_{\mathrm{BP}}\left(0\right)=\epsilon_{\mathrm{BP}}$ and *R* are the BP threshold and design rate of the (unpunctured) mother code ensemble, respectively, and $R(\alpha_{p})$ is the target rate after puncturing.

**Figure 1:**
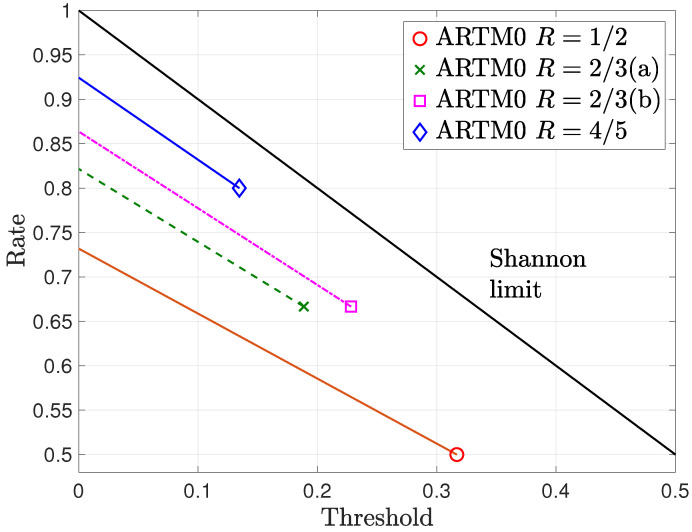
BEC BP thresholds of randomly punctured ARTM0 LDPC code ensembles for a variety of puncturing fractions $\alpha_{p}$.

Figure 1 shows numerically calculated BP thresholds of the randomly punctured ARTM0 LDPC code ensembles for a variety of puncturing fractions α. The mother LDPC code ensemble thresholds $\epsilon_{\mathrm{BP}}\left(0\right)$ are shown by markers (circle, cross, square, and diamond), with the corresponding punctured thresholds $\epsilon_{\mathrm{BP}}\left(\alpha_{p}\right)$, for various values of $\alpha_{p}$ and corresponding rate $R(\alpha_{p})$, shown by the lines from a given marker. We observe relatively robust performance under puncturing, particularly for the R=2/3(b) and R=4/5 ensembles, for which $\epsilon_{\mathrm{BP}}\left(0\right)$ begins closer to capacity. For each ensemble, the threshold values decrease with increasing $\alpha_{p}$ as $R(\alpha_{p})$ increases. Since none of the curves cross, this indicates that random puncturing beyond the rates of the next mother code ensemble is unlikely to provide any advantage in terms of performance. We also note that the rate for which the lines reach a 0 value of the threshold is indicative of the maximum rate achievable for random puncturing of the given ARTM0 LDPC code ensemble.

### 3.2. Minimum Distance Growth Rates of Randomly Punctured ARTM0 Code Ensembles

A code ensemble is called *asymptotically good* if the minimum distance typical of most members of the code ensemble is at least as large as $\delta_{\min}\cdot n$, where $\delta_{\min}>0$ is called the *minimum distance growth rate* of the ensemble. To test this definition, the *asymptotic spectral shape* of a code ensemble, defined as

(8) $$r\left(\delta \right)=\underset{n\to \infty }{lim sup}\frac{1}{n}\ln\left(A_{\left\lfloor \delta n\right\rfloor }\right),$$

can be used, where δ=d/n is the normalized Hamming distance, n∈N is the block length, and $A_{d}$ is the ensemble weight enumerator. For protograph-based LDPC code ensembles, a method to determine r(δ) was presented in [24]. For J>2, (J,K)-regular LDPC codes are known to be asymptotically good [25]. As a result of the variable node degree optimization of the ARTM0 protographs, we find that these ensembles are asymptotically good, with the computed minimum distance growth rates shown in Table 1.

For an asymptotically good code ensemble with spectral shape r(δ), the expected asymptotic spectral shape of the randomly punctured code ensemble can be characterized as [26]

(9) $$\begin{array}{c} r^{\mathrm{punc}}\left(\delta \right)=\frac{1}{1-\alpha_{p}}\left(\max_{0\leq \lambda \leq 1}\left\{\lambda \cdot h\left(\frac{(1-\alpha_{p})\delta }{\lambda }\right)+\right.\right.\\ \;\;\;\;\;\;\;\;\;\;\;\;\;\;\;\;\;\;\;\;\;\;\;\;\;\;\;\;\;\;\;\;\;\;\;\;\left.\left.\left(1-\lambda \right)\cdot h\left(\frac{\alpha_{p}+\left(1-\alpha_{p}\right)\delta -\lambda }{1-\lambda }\right)+r\left(\lambda \right)\right\}-h\left(\alpha_{p}\right)\right), \end{array}$$

where $\alpha_{p}=p/n$ is the fraction of punctured bits, $0\leq \alpha_{p}<\delta_{\min}$, and h(δ)=−(1−δ)ln(1−δ)−δln(δ) is the binary entropy function. The average weight enumerators used in the formulation of (9) are obtained over all possible *p*-bit puncturing patterns; therefore, we require $\alpha_{p}<\delta_{\min}$ to guarantee no rate loss. For $\alpha_{p}\geq \delta_{\min}$ the rate of the ensemble can be written as $\left(R-\Delta_{R}\right)/\left(1-\alpha_{p}\right)$, where $\Delta_{R}\geq 0$. Bounds on $\Delta_{R}$ and conditions such that $\Delta_{R}=0$ were given for (J,K)-regular LDPC code ensembles in [27].

**Figure 2:**
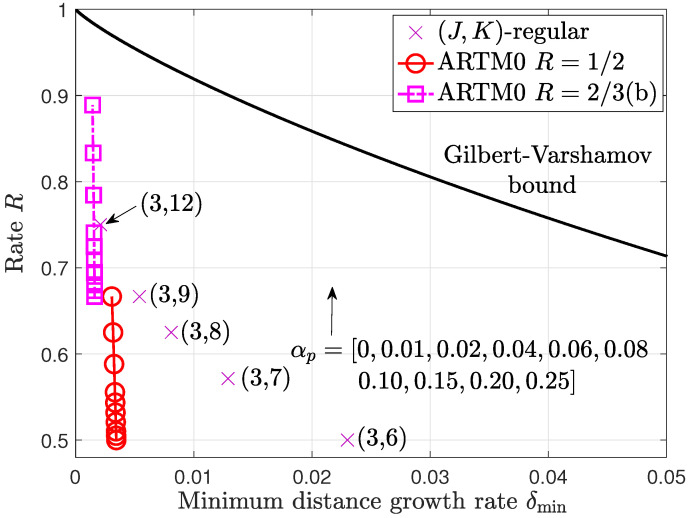
Minimum distance growth rates for the punctured ARTM0 LDPC code ensembles with a variety of puncturing fractions $\alpha_{p}$. The arrow indicates the direction of increasing $\alpha_{p}$ corresponding to the point markers on the ARTM0 curves.

In Figure 2, we present numerical results for the ARTM0 R=1/2 and R=2/3(b) ensembles obtained using (9) for a variety of puncturing fractions $\alpha_{p}$. We note that, in most cases, $\alpha_{p}>\delta_{\min}$, and, as a consequence, there can be some rate loss where the true rate is bounded above by $R(\alpha_{p})$. In practice, the bits to be punctured, randomly or otherwise, can be selected to avoid a rate loss by preserving the dimension of the code. Therefore, the numerical results in this section are a useful tool to gauge the performance of the punctured code in practical scenarios.

We observe that, like the mother code ensembles, the punctured ARTM0 LDPC code ensembles are also asymptotically good. Although the minimum distance growth rates must decrease with $\alpha_{p}$, the ARTM0 growth rates show robust behavior as the code rate increases, where we observe that the distance to the Gilbert–Varshamov bound decreases with $\alpha_{p}$. In particular, the punctured R=2/3 ensemble with $\alpha_{p}=0.1$ has a similar growth rate as the (3,12)-regular LDPC code ensemble. Taken along with the iterative threshold analysis results from Section 3.1, it appears that the ARTM0 LDPC code ensembles are indeed good candidates for random puncturing.

## 4. Random Puncturing of ARTM0 LDPC Codes

### 4.1. System Model

**Figure 3:**
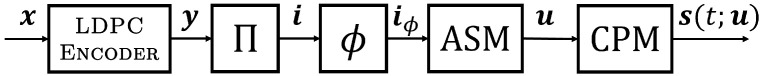
Model transmitter for punctured LDPC code with CPM modulation.

The transmitter model utilized in our system is shown in Figure 3, with function blocks representing the LDPC encoder, interleaver (Π), puncturing (ϕ), CPM modulator, and a module that allows for the insertion of a known symbol sequence, referred to as an attached synchronization marker (ASM) which helps to identify the beginning of each codeword c at the receiver. LDPC encoding is performed by multiplying the source information sequence x of length *k* with the (systematic, in the case of the ARTM0 codes) code generator matrix G of size k×n to form codeword y=xG. The resulting codeword y is interleaved to form the sequence i, on which puncturing is performed. Note that puncturing is placed after interleaving so that a single interleaver of a fixed length may be used throughout the entire system model. Given a specific interleaver, puncturing can be achieved in a rate-compatible way by varying the amount and location of the punctured bits.

The resulting punctured codeword, $i_{\phi }$, is concatenated with the ASM to form a frame u, which serves as the modulating sequence of the CPM, whose output is sent over the communication channel as the sequence of q-ary frames s(t;u) where *q* is the modulation order. Puncturing (ϕ) must be performed at the encoder prior to the concatenation of the interleaved codeword and ASM in order to avoid desynchronization during decoding. In this paper, to demonstrate the average performance of the code-modification techniques, the puncturing and interleaving patterns are drawn uniformly from a random distribution. However, specific and optimized patterns could be achieved in practice with a simple mapping of the fixed locations (e.g., certain parity symbols only) of the target symbols to their fixed interleaved locations.

Efficient LDPC encoding algorithms are suitable for many low-power, memory-constrained applications [7,16]. This is true for the ARTM LDPC codes we have used, which have a quasi-cyclic (QC) structure in which the code’s parity check matrix is an array of sparse repeating units called *circulants* [4]. The LDPC code utilized in many 5G wireless networks is also a protograph-based, quasi-cyclic code, and the techniques we describe have been studied to provide rate compatibility under the constraints of that standard [15]. The most commonly implemented decoder for LDPC codes is a special case of a more general BP algorithm used for inference on graphical models, known as the sum-product algorithm (SPA) [28]. In this paper, we have used SPA decoding because it provides good performance with soft-input–soft-output (SISO) functionality. Efficient hardware implementations of such decoders are numerous and widely adopted [6], including reduced-complexity variants such as the min-sum algorithm (MSA) [7].

**Figure 4:**
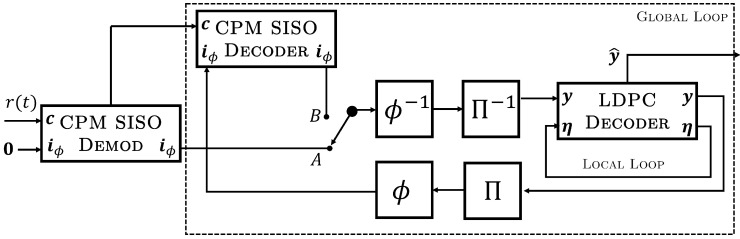
Model receiver for punctured LDPC code with CPM modulation and iterative decoding.

The receiver model is shown in Figure 4. In this diagram, the values passed between blocks are all real-valued vectors, i.e., log-likelihood ratios (LLRs) [19], that correspond to the discrete-valued sequence indicated at the input/output of the function blocks. Inputs are always *a priori* information, while outputs are *a posteriori* information, with a posterori information from each decoder serving as *a priori* input to the decoder at the next time increment. Since the signals in our diagram reference the registers used to store soft information vectors during processing, they are reused for both inputs and outputs to provide a functional description rather than signal isolation that may be required in hardware. The received sequence r(t), corresponding to a CPM word c, is input to the CPM demodulator along with *a priori* information on $i_{\phi }$ (the binary interleaved and punctured codeword from the LDPC code) that is initialized to zero. After the demodulation of a codeword, it is decoded iteratively in a global loop by a pair of CPM and LDPC decoders, which exchange *a priori* information on $i_{\phi }$ through an interleaver Π and puncturing block ϕ, identical to those used for encoding, with corresponding blocks for the inverse deinterleaver $\Pi^{-1}$ and the depuncturer $\phi^{-1}$ (explained below). For the first global iteration, the results of the CPM demodulator are permuted and used as soft-valued inputs (channel LLRs) for the local LDPC decoder loop by placing the switch in position A. For subsequent global iterations, the signal flow is altered by placing the switch position in B, with the soft output LLRs of the CPM SISO decoder’s update now serving as *a priori* inputs to the LDPC decoder. Note that we have separated the CPM receiver circuit into two modules: the CPM demodulator and the decoder. This is a practical choice since the CPM demodulator needs to act only once on a received block, where certain signal processing functions, such as filtering, are unnecessary within the global iterative decoding loop. In this context, the CPM SISO decoder refers specifically to the trellis decoder within the CPM receiver which provides additional soft information to the SPA decoder.

LDPC SPA decoding is performed using the code’s parity-check matrix H, a sparse matrix that satisfies the condition $GH^{T}=0$. Sparsity keeps the memory and computational requirements tractable for coding schemes with long block lengths that are needed to obtain capacity-approaching performance. The rows of H correspond to the parity check conditions of the code, while each column corresponds to a codeword symbol. If a one is present in the *k*-th column of row *j*, code symbol *k* is found in the parity check equation $A_{j}$. The LDPC decoder performs iterative SPA decoding in the local loop before passing the output LLRs on y as *a priori* information on $i_{\phi }$ to the CPM SISO decoder to start the next global iteration, and so on. We note that in our implementation, the LDPC decoder produces soft *a posteriori* information and retains those values as *a priori* information on the punctured symbols for the next iteration within the local loop. This is indicated in Figure 4 by the signal path η, which is initialized to zero prior to the first global iteration. The process continues until some stopping criterion is reached (such as a ceiling on the allowed number of iterations) and the resulting estimated codeword $\hat{y}$ is passed out of the decoder. Upon completion, hard decisions on $\hat{y}$ are made to complete decoding, where $\hat{x}$ is directly accessible since the encoder is systematic.

**Figure 5:**
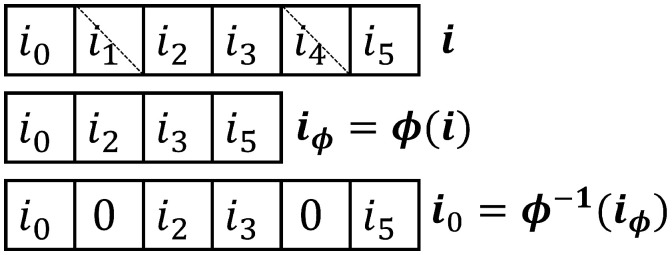
Puncturing by truncation and depuncturing by zero-padding within the global decoding loop of the receiver.

Puncturing is performed in the global loop between the CPM SISO decoder and the LDPC decoder. The module ϕ removes codeword soft information symbols in accordance with the known puncturing pattern set by the transmitter, resulting in a truncated sequence $i_{\phi }$, as shown in Figure 5. The module $\phi^{-1}$ intercalates zeros into these same puncturing positions to expand the sequence to its original length before interleaving, in a process referred to as *depuncturing*. Since the messages passed in the SPA correspond to LLRs, the zero-valued LLRs that are placed into the puncturing positions correspond to a bit being equally likely a one or a zero. Finally, we note that at the receiver (Figure 4), the puncturing, depuncturing, interleaving, and deinterleaving all operate on real-valued vectors.

For the block code we are implementing, the code rate after puncturing, $R(\alpha_{p})$, is determined solely by the mother (unpunctured) code rate and puncturing overhead, as given in (5). Therefore, the amount of overhead required for any desired code rate is $\Delta =\left(1-R/R(\alpha_{p})\right)\cdot 100\%$. The codes outlined in [9] are *systematic* LDPC codes, wherein the source information sequence appears unaltered in the encoded sequence, but in general, this is not a requirement because no distinction is made between information and parity symbols in our scheme and puncturing is performed after interleaving. These codes exhibit sufficient regularity such that information symbols and parity symbols are equally protected and punctured symbols are therefore not limited to parity bits in our study.

### 4.2. Numerical Results

In this section, we present the results of our numerical simulations which were performed on the additive white Gaussian noise (AWGN) channel with BPSK and CPM modulation. For the CPM simulations, we followed [9] and set a maximum of 512 global iterations and a maximum of one LDPC decoder iteration with a syndrome-based stopping rule. For the LDPC-code-only simulations, we used BPSK modulation and set a maximum of 100 LDPC decoder iterations and a syndrome-based stopping rule. Although computationally inequivalent, we have chosen these parameters to represent the best case scenario in terms of FEC performance for each configuration, since LDPC decoding is enhanced by information from the CPM decoder and we wish to maximize information exchange between the component decoders [29]. In practice, the choice of stopping criteria would depend on computational and/or latency constraints at the receiver [30], which we chose not to consider in these experiments since it does not directly impact spectral efficiency. To obtain an estimate of the average random puncturing performance, we used a different random puncturing pattern per transmitted codeword in our simulations. Notationally, we now add a subscript corresponding to the mother code rate to distinguish the codes; e.g., $R_{2/3}\left(0.1\right)$ denotes the rate of the punctured code obtained from the rate $R_{2/3}\left(0\right)=R=0.667$ mother code with $\alpha_{p}=0.1$, or 10% puncturing. Note that, in this paper, rates are rounded to three decimal places.

#### 4.2.1. BPSK Modulation

**Figure 6:**
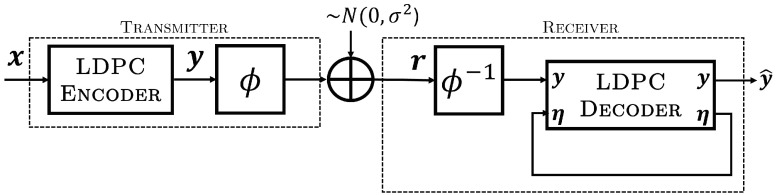
Simplified transceiver model for BPSK modulation.

**Figure 7:**
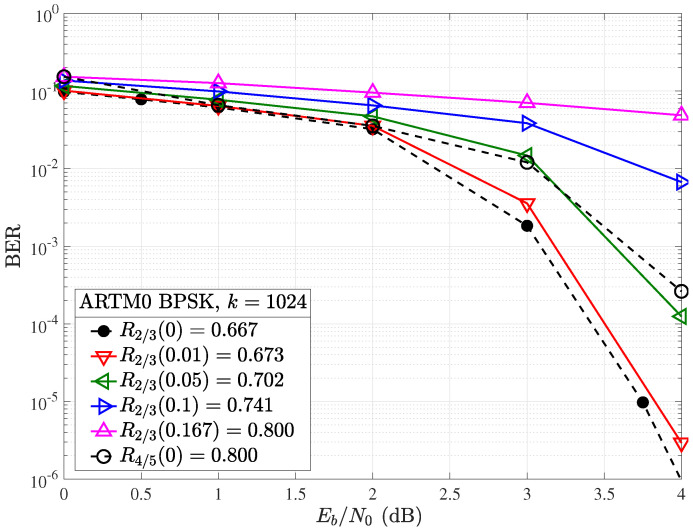
BER performance with BPSK modulation for the R=2/3, k=1024 ARTM LDPC code with (solid) and without (dashed) random puncturing.

In order to isolate the effect puncturing has on the decoding performance of the LDPC code alone, we have removed the CPM and associated support modules, utilizing only BPSK modulation, which results in the greatly simplified transceiver model shown in Figure 6. With this model, puncturing is performed immediately before encoded information is sent over the communications channel and depunctured after being received only once. Due to the elimination of the global iterative loop used to exchange information between the CPM and LDPC decoders (c.f., Figure 4), the depunctured information (soft-valued channel LLRs) directly enters the SPA LDPC decoder, where it is iteratively decoded to produce codeword estimate $\hat{y}$. Simulated error correction performance on the AWGN channel for the R=2/3 ARTM0 code with information block length k=1024 and puncturing overhead values of Δ=(0%,1%,5%,10%,16.7%) is shown in Figure 7. The k=1024, R=2/3, ARTM0 code is constructed from the lifted base matrix $B_{0b,2/3}$ [9]. The value Δ=16.7% is the percentage of puncturing necessary to create a $R_{2/3}\left(0.167\right)=0.8$ code from a R=2/3 mother code.

Without leveraging CPM decoder information, decoding is strongly affected by random puncturing, with 0.5 dB of coding gain lost at a bit error rate (BER) of $10^{-4}$ when moving from Δ=0 to Δ=5, which represents an effective rate increase from R=2/3 to $R_{2/3}\left(0.05\right)=0.702$. Although small values of puncturing are well tolerated, such as shown for Δ=1%, these do not provide a significant increase in the code rate (e.g., Δ=1% increases R=2/3 to $R_{2/3}\left(0.01\right)=0.673$). It is also observed that puncturing to the next available code rate of R=4/5 by setting Δ=16.7% results in poor performance, since the decoder cannot compensate for this large Δ. Although it may happen that a code optimized for use with CPM is suboptimal for SPA decoding, it is worth noting that a coding gain of over 7 dB was reported [10] with the modulation scheme in place, outperforming all existing codes that were not optimized for the same modulation schemes.

#### 4.2.2. CPM Regime

**Figure 8:**
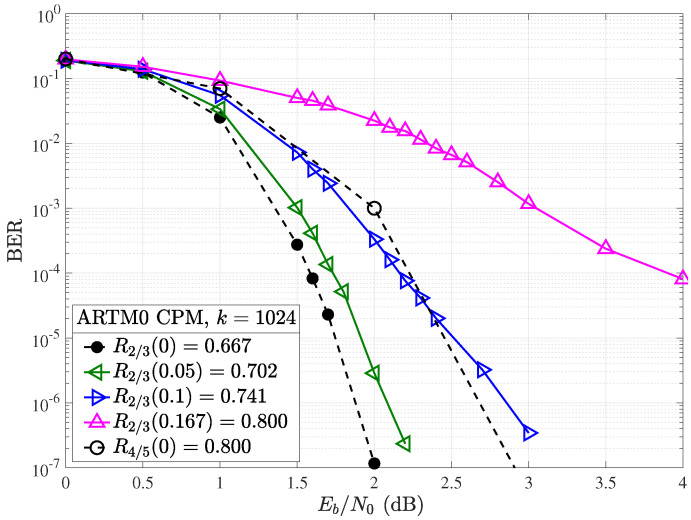
BER performance with CPM for the R=2/3, k=1024 ARTM LDPC code with (solid) and without (dashed) random puncturing.

With CPM included, as shown in the systems presented in Figure 3 and Figure 4, a significant coding gain can be attributed to the global decoder-modulation loop. As shown in Figure 8, a coding gain in excess of 2 dB is observed between the systems employing BPSK and CPM modulation schemes, even without considering puncturing (Δ=0%). This illustrates the chosen LDPC code’s optimization for ARTM modulation and the ability of the SPA decoder to take advantage of information provided by the CPM decoder, working in concert by successively exchanging more accurate *a priori* information in a doubly iterative fashion.

For the full system, puncturing has a much more gradual influence on the knee or *waterfall* region of the resulting BER curve, with an error floor observed above a BER of $10^{-7}$ only for overheads in excess of 10%. For example, with Δ=5%, corresponding to an increase of the code rate from R=2/3=0.667 to $R_{2/3}\left(0.05\right)=0.702$, we observed a loss of approximately 0.5 dB at a BER of $10^{-4}$ in the simplified BPSK case (Figure 7), whereas in the CPM case, we see that this is reduced to approximately 0.1 dB. At a bit error rate of $10^{-6}$, the loss remains less than 0.2 dB. At Δ=10%, with $R_{2/3}\left(0.1\right)=0.741$, we see the punctured code still has reasonable performance (unlike BPSK). At this overhead, it performs similarly to the next highest available rate code of R=4/5, indicating that, at this point, it may be preferable to use the next code in the standard for higher rates rather than puncture the lower rate codes. R=4/5 is not achieved until Δ=16.7%, at which point the performance dramatically degrades (like the case with BPSK). This occurs as a result of the LDPC decoder failing to converge satisfactorily. However, we conjecture that optimized puncturing patterns [31,32] may be able to reduce this gap. This is the subject of ongoing research.

**Figure 9:**
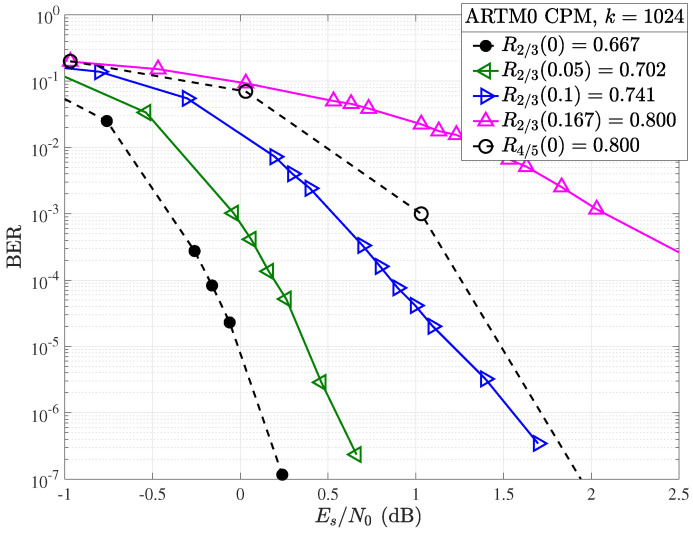
BER performance in terms of $E_{s}/N_{0}$ with CPM for the R=2/3, k=1024 ARTM LDPC code with (solid) and without (dashed) random puncturing.

In order to better examine the region of interest around the R=4/5 target code, Figure 9 displays the relevant curves across the punctured R=2/3 codes along with the unpunctured R=4/5 modulated ARTM code in terms of the ratio of energy per symbol to the single-sided noise power spectral density $E_{s}/N_{0}$. The gain between the punctured Δ=10%, $R_{2/3}\left(0.1\right)=0.741$ code and unpunctured mother R=4/5 code is approximately 0.4 dB at a BER of $10^{-4}$, approximately the same as the loss in the gain when going from the mother R=2/3 code to punctured Δ=5%, $R_{2/3}\left(0.05\right)=0.702$ code, highlighting that a window for further improvements is viable. We see, however, that the gap between the punctured $R_{2/3}\left(0.1\right)=0.741$ code and the mother $R_{4/5}\left(0\right)=0.8$ code has decreased to approximately 0.15 dB at a BER of $10^{-6}$ and looks likely to cross at higher SNR since the punctured waterfall is not as steep as the higher rate mother code. Indeed, this error floor behavior continues for Δ≥10%. See, for example, the $R_{2/3}\left(0.167\right)=0.800$ code, which has a significant error floor and loss of over 1dB (and increasing) for BERs lower than $10^{-3}$ when compared to the mother $R_{4/5}\left(0\right)=0.8$ code.

The simulation results presented previously [10] confirm the formation of an error floor below a BER of $10^{-6}$ for the R=2/3 mother code, perhaps indicating an underlying weakness in that particular code that is exacerbated by puncturing irrespective of the modulation scheme. At low values of overhead Δ≤5%, no error floor is yet apparent at $10^{-6}$, and thus, error-floor effects seem to dominate only in high overhead regimes. This gives possible allowance for low-overhead random puncturing to be realizable without any further considerations necessary. We also note that low-overhead puncturing where Δ≤10% is well tolerated, and performance is robust with only a gradual decrease in the slope of the BER curve within the waterfall region. Operation within the waterfall region is nevertheless practical and could serve as an adaptive mechanism when spectral bandwidth is limited and channel conditions are favorable.

## 5. Random Shortening of ARTM0 LDPC Codes

As described in Section 2.3, shortening is a complementary technique to puncturing wherein codes of shorter length and lower rate can be constructed from a higher-rate mother code. To accomplish this in our proposed rate-compatible architecture, infinite reliabilities are assigned for the selected (shortened) information bits whose positions are known to both the transmitter and receiver, removing those bits from the resulting codeword. This corresponds to the removal of the matching information columns from the LDPC code’s parity-check matrix, reducing the code rate as given in (6).

### 5.1. Model and Method

**Figure 10:**
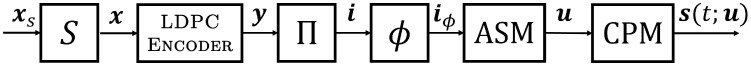
Modification to the transmitter circuit includes module *S* to shorten the information bit sequence.

The system model for shortening is nearly identical to that provided in Section 4.1 for puncturing (see Figure 3 and Figure 4), with the only alteration being to the function blocks ϕ and $\phi^{-1}$ and the inclusion of a function block S that is used to shorten the information sequence. In the shortening transmitter model (Figure 10), module S takes k−s input information bits as the input sequence, $x_{s}$, and inserts *s* zeros in fixed (shortening) positions, thereby producing an information sequence x of length *k*, which serves as input to the LDPC encoder. Since the encoder is systematic, the corresponding code symbols will also be zero, as prescribed by our shortening model. Note also that this excludes any parity bits from being shortened (fixed to zero) and effectively removes the corresponding rows from the generator matrix utilized by the LDPC encoder.

**Figure 11:**
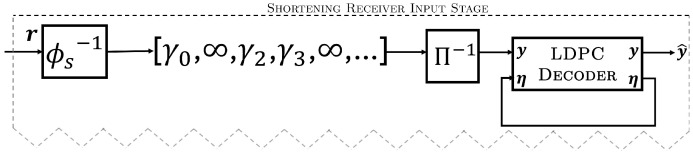
Modifications to the input stage of the decoder in Figure 4 showing the $\phi_{s}^{-1}$ module producing the infinite LLR outputs required for shortening.

In the shortening transmitter and receiver models, ϕ operates in a similar way to puncturing but now only removes the known (shortened) information bits of the code (after interleaving). With puncturing, $\phi^{-1}$ operates to insert zero-valued LLRs, corresponding to an equal probability of a bit being a zero or one since this information is not shared between the transmitter and the receiver prior to transmission (but must still be estimated). However, in the shortening receiver model (see Figure 11), module $\phi_{s}^{-1}$ operates by inserting *infinite* valued LLRs (practically, a very large positive value) in the exact positions corresponding to the shortening pattern selected by the transmitter. This ensures the shortened symbols are fixed to zero, removing them from the parity check equations performed by the LDPC decoder. Similar to puncturing, we will consider various values of shortening overhead $\alpha_{s}=s/n$, as defined in Section 2.3, or as a percentage $\Delta_{s}=\alpha_{s}\cdot 100\%$. We denote the rate of the shortened code with a subscript according to the rate of the mother code; e.g., $R_{2/3}^{s}\left(0.1\right)$ denotes the rate of the shortened code obtained from the rate $R_{2/3}^{s}\left(0\right)=R=0.667$ mother code with $\alpha_{s}=0.1$.

### 5.2. Numerical Results

**Figure 12:**
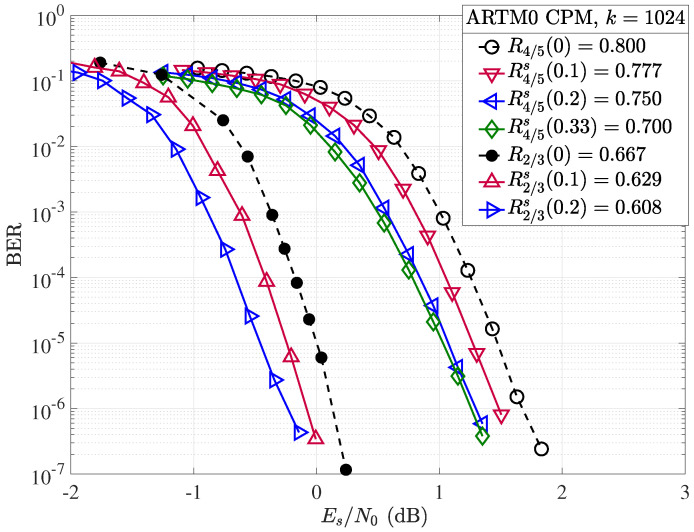
Random shortening applied to the ARTM0 R=2/3 and R=4/5 codes in terms of $E_{s}/N_{0}$.

**Figure 13:**
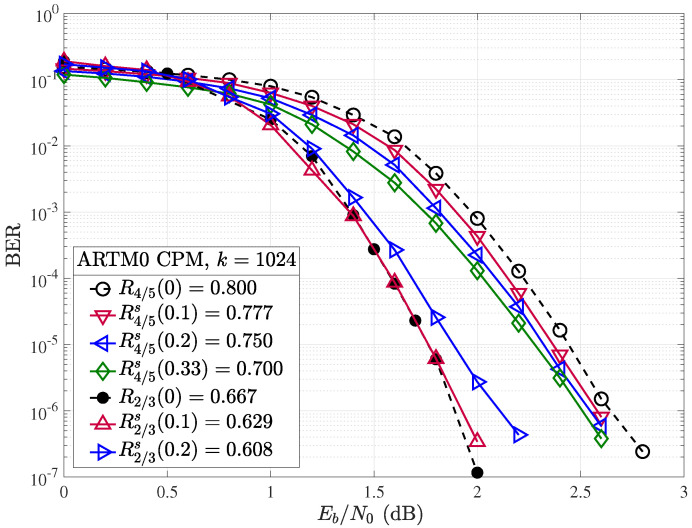
Random shortening applied to the ARTM0 R=2/3 and R=4/5 codes in terms of $E_{b}/N_{0}$.

Simulation results were obtained with the same experimental setup used for random puncturing, as described in Section 4.2, with the exception that a different random shortening pattern is selected per transmitted codeword. Both the rate and block length of the mother code play an important role in the performance of ARTM0 codes under random shortening. If we first consider the performance of the shortened k=1024, R=2/3(b), and R=4/5 codes in terms of $E_{s}/N_{0}$ (Figure 12), we observe an improvement in performance with increasing $\Delta_{s}$ (and lowering rate $R^{s}\left(\alpha_{s}\right)$), as expected. However, when we adjust for the rate and consider the same examples in terms of the ratio of energy per bit to the single-sided noise power spectral density $E_{b}/N_{0}$ (Figure 13), no gain is observed by shortening the mother R=2/3 code with $\Delta_{s}=10\%$, and a loss is observed as we further increase to $\Delta_{s}=20\%$. However, with the higher rate of R=4/5 mother code, the larger gains observed in $E_{s}/N_{0}$ in Figure 12 do translate to improvements in terms of $E_{b}/N_{0}$. We observe that each 10% step increase in shortening from $\Delta_{s}=10\%$ to $\Delta_{s}=20\%$ produces an approximate gain of about 0.05 dB per step at a BER of $10^{-5}$. We remark that with $\Delta_{s}=40\%$ (not shown), the shortened code rate is $R_{4/5}^{s}\left(0.4\right)=2/3$, but the performance falls short of the R=2/3 mother code. Similar results to those of the R=2/3 code are obtained for the shortened R=1/2 codes, where gains are observed in terms of $E_{s}/N_{0}$, but not when correcting for the rate with $E_{b}/N_{0}$. These results indicate the short (k=1024) ARTM0 block codes of low rates are less amenable to shortening, making rate-matching difficult for lower-rate mother codes but possible with the higher-rate mother codes. As with the random puncturing, it is possible that specific shortening patterns could yield gains over random shortening.

**Figure 14:**
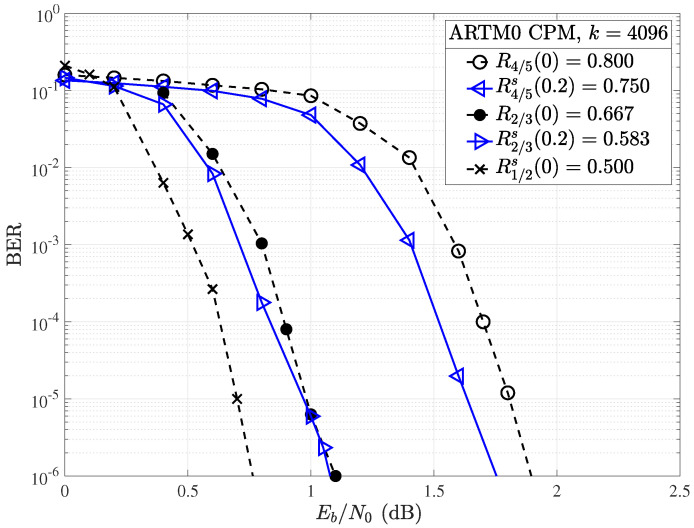
Random shortening applied to the ARTM0 R=4/5 and R=2/3 codes with k=4096.

If larger block lengths are considered, shortening produces better results in terms of overall BER performance, especially for higher-rate codes. As shown in Figure 14, a coding gain of 0.2 dB is observed in the waterfall region of the R=4/5 code with information block length k=4096 and $\Delta_{s}=20\%$, whereas a gain of approximately 0.1 dB was observed for the code with k=1024 of the same rate and the same amount of shortening. This implies that longer block codes may be more amenable to random shortening, making rate-matching easier to accomplish with acceptable losses in performance.

For both information block lengths considered, the code with a mother rate R=4/5 shortened by $\Delta_{s}=20\%$ produces a code of rate R=3/4=k/(k+1), providing a convenient rate-compatible option for framing and hardware implementation. For the R=2/3 mother code, moving to a larger information block length of k=4096 does produce a slight gain at $R_{2/3}^{s}\left(0.2\right)=0.583$ within a narrow portion of the waterfall region, which was not observed for random shortening of the corresponding k=1024 code. Even with the longer block lengths, random shortening may have a negative impact on performance when adjusting for rate.

### 5.3. Puncturing vs. Shortening

**Figure 15:**
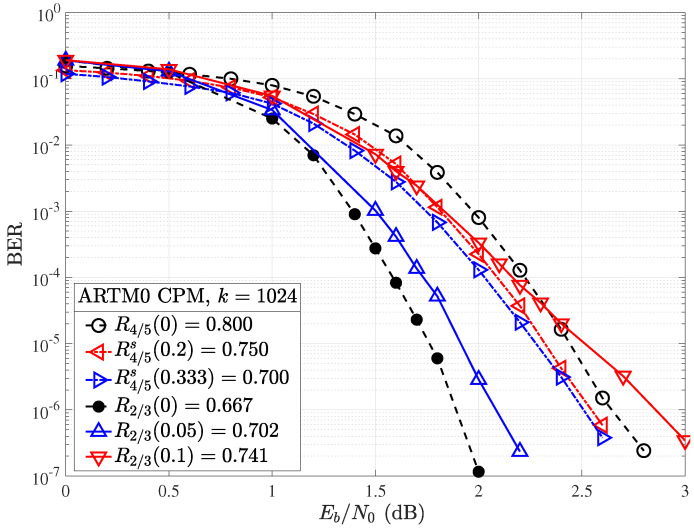
BER performance for the R=2/3, k=1024 ARTM LDPC code with random puncturing (solid) alongside the R=4/5, k=1024 ARTM LDPC code with random shortening (dot-dash).

Finally, we briefly discuss the trade-offs observed by puncturing a lower-rate ARTM0 code verus shortening a higher-rate ARTM0 code. Figure 15 shows the results obtained for random puncturing of the R=2/3, k=1024, code with Δ=5 and 10% (solid lines) alongside those obtained for random shortening of the R=4/5, k=1024, code with $\Delta_{s}=20$ and 33% (dot-dash lines) with a goal of hitting target rates of approximately 0.7 (blue) and 0.75 (red). We observe that for rates close to the lower rate ARTM0 code, puncturing offers the best performance, whereas for the higher rates between the mother codes, it may be preferable to shorten the higher-rate code. For example, to obtain R=0.7, we find that it is a better strategy to puncture the R=2/3 mother code, whereas for a target rate of R=3/4, we find that it is better to shorten the mother R=4/5 code.

## 6. Hardware Considerations

Random puncturing and shortening may be implemented by the insertion of a switch in the registers that serve as buffers to the inputs of each module in the signal chain. At random intervals chosen by the overhead parameters, block length, and system bus speed, the switch is placed in the high-impedance position for one clock cycle. Depuncturing/deshortening is accomplished by the insertion of a random delay, at which point a zero-value (puncturing) or infinite value (shortening) is inserted into the register. Although this introduces additional latency, because all modules operate blockwise, it is possible to perform both puncturing and depuncturing in parallel.

Thus, when operating in a low-overhead regime as our results suggest, random puncturing and shortening offer robust rate-compatible performance with no changes to the submodules of Figure 3 and Figure 4. Since no substantive hardware alterations are required, this allows for new operational modes at little expense in terms of latency and memory. However, it is worth noting that because the LDPC SPA decoder still operates on the full depunctured/deshortened sequence, SPA-decoding computational complexity will remain largely unchanged.

## 7. Conclusions

In this paper, we have laid the groundwork for the construction of rate-compatible LDPC codes for IRIG-106 waveforms by the use of random puncturing and shortening. Our initial results show robust performance with codes designed for deployment with ARTM CPM modulation and open promising avenues for further improvement. Such systems would allow for the adaptive allocation of bandwidth in response to changing demand or channel conditions without requiring significant changes to the underlying encoding and decoding hardware and thus represent a low-complexity, low-cost implementation option. Furthermore, because puncturing is a type of erasure channel, it is easily modeled, and those results are readily extended to other channel models, reducing design iteration schedules.

By isolating the LDPC encoder and decoder, we were able to show the considerable coding gain afforded by the CPM-LDPC decoder loop, reinforcing that coding systems designed specifically for these modulations are better optimized than those that do not consider modulation during design. Though in neither case did we observe that random puncturing alone achieves the same performance as a code designed for that specific target rate, the gap is much more tractable with CPM modulation, having a span of 0.5 dB, whereas without CPM modulation, the required overhead elicits an impractical error floor. Shortening k=4096 codes with CPM modulation is a viable method for producing rates in between the mother code rates, but the technique works best for shortening the highest rate R=4/5 code with diminishing returns for lower-rate mother codes.

In future work, other codes, including those of different rates and lengths, will be considered in order to better understand the interplay between the strength of the code and its resilience to puncturing and shortening under CPM. Modulation appears to be no impediment to the implementation of puncturing, with perturbations in the codeword’s structure most strongly felt within the inner LDPC decoding loop of Figure 4. Because the code we are using in our model is designed specifically to take advantage of information from the CPM decoder, further work should examine the selection of puncturing patterns that least disturb this feedback mechanism in order to preserve its portion of the total coding gain, which was observed to be in excess of 2 dB. Furthermore, analytical results from density evolution on the BEC indicate not only that preferential puncturing patterns exist but that catastrophic patterns exist that yield extremely low decoding thresholds and should be avoided [13]. This knowledge will help inform the selection of nonrandom or quasi-random puncturing patterns that may bridge the gap between the punctured and mother codes seen in our present analysis. It will also better describe the limits of the code design procedure and may offer new avenues for optimization.

## Figures and Tables

**Table 1:** Minimum distance growth rates for the ARTM0 LDPC code ensembles.

ARTM0	R=1/2	R=2/3(a)	R=2/3(b)	R=4/5
$\delta_{\min}$	0.00344	0.00066	0.00161	0.00032

## Data Availability

Data is contained within the article.

## Abbreviations

The following abbreviations are used in this manuscript:

ARTM	Advanced Range TeleMetry
ASM	Attached Sync Marker
AWGN	Additive White Gaussian Noise
BEC	Binary Erasure Channel
BER	Bit Error Rate
BP	Belief Propagation
BPSK	Binary Phase-shift Keying
CPM	Continuous Phase Modulation
FEC	Forward Error Correction
FM	Frequency Modulation
LDPC	Low-Density Parity-Check
LLR	Log-likelihood Ratio
MSA	Min-sum Algorithm
PCM	Pulse Code Modulation
QC	Quasi-cyclic
SISO	Soft-in, Soft-out
SOQPSK-TG	Telemetry Group version of Shaped-Offset Quadrature Phase Shift Keying
SPA	Sum-product Algorithm
